# Rapid Prototyping of cardiac models: current utilization and future directions

**DOI:** 10.1186/1532-429X-14-S1-T13

**Published:** 2012-02-01

**Authors:** Omar Thabit, Shi-Joon Yoo

**Affiliations:** 1 Diagnostic Imaging, The Hospital for Sick Children, Toronto, ON, Canada

## Summary

Rapid 3D-Prototyping is an established technique that converts digital image data of any 3D structure to a physical 3D model. It has been used for a long time in industry for making prototypes of any new products. The state-of-the-art medical imaging facilities such as computed tomography and magnetic resonance imaging (and possibly echocardiography in the future) provide precise digital information of the cardiovascular structures of the human body. The digital information can be used for production of multiple replicas of the human body parts with solid or flexible materials. Virtual visualization of 3D information in the computer screen has revolutionized medical imaging in the last 10-20 years. Although virtual visualization facilitates understanding, it does not allow direct contact or manipulation on the physical model. 3D prototyping of the replicas certainly allows direct visual access to the physical structures and more importantly direct physical manipulation such as practice surgery on the replica of the structure to be operated. The models are excellent teaching materials to all involved in cardiac imaging or surgery. Production of 3D prototypes of various pathologic conditions is even more important as there has been increasing restrictions to keeping human body parts for teaching as well as clinical purposes and pathologic specimens are available only when they are removed at surgery or at autopsy.

## Background

Rapid 3D-Prototyping is an established technique that converts digital image data of any 3D structure to a physical 3D model. It has been used for a long time in industry for making prototypes of any new products.

## Methods

The state-of-the-art medical imaging facilities such as computed tomography and magnetic resonance imaging (and possibly echocardiography in the future) provide precise digital information of the cardiovascular structures of the human body. The digital information can be used for production of multiple replicas of the human body parts with solid or flexible materials.

## Results

We have experienced the utility of 3D plastic models in preoperative assessment of the cases with double outlet right ventricle in the last two years,and are convinced that this technology should be further expanded for the following purposes:

1)To produce heart models for simulations of surgical procedures in patients with complex surgical anatomy.

2)To produce simulated phantoms of various surgical pathways such as Fontan circuit and right ventricle-to-pulmonary artery conduit for assessment of flow through complicated surgical pathways.

3)To produce a collection of teaching models for normal and pathological cardiovascular anatomy.

4)To develop a technique of image fusion for complete prototyping by using data from multiple imaging modalities.

## Conclusions

Virtual visualization of 3D information in the computer screen has revolutionized medical imaging in the last 10-20 years. Although virtual visualization facilitates understanding, it does not allow direct contact or manipulation on the physical model. 3D prototyping of the replicas certainly allows direct visual access to the physical structures and more importantly direct physical manipulation such as practice surgery on the replica of the structure to be operated.

**Figure 1:**
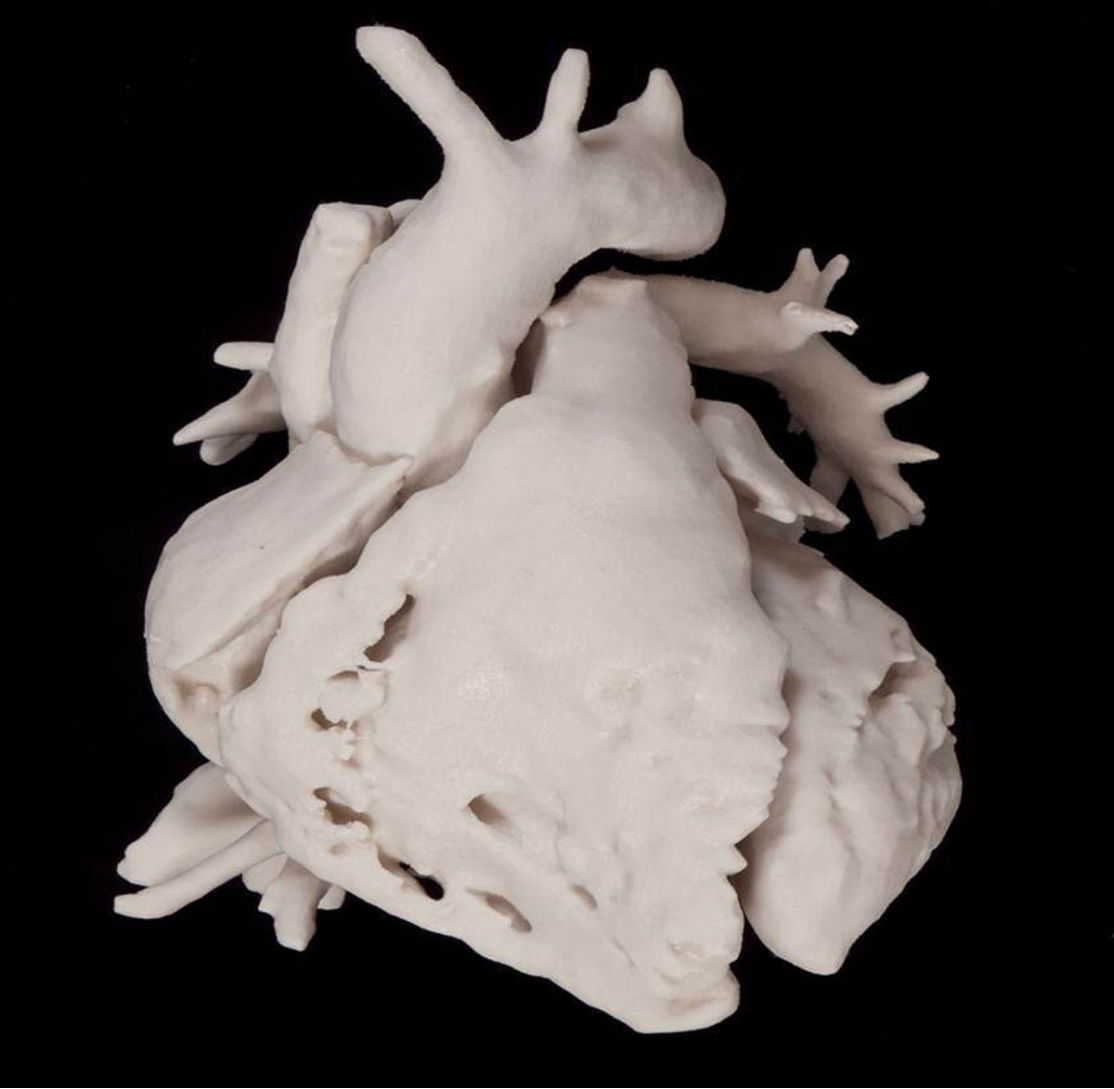
Cast model of normal heart.

**Figure 2:**
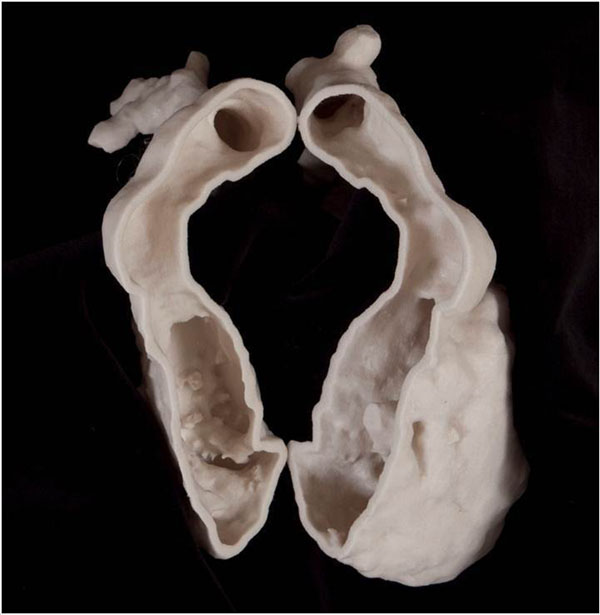
Cavity model of the right ventricle showing stenotic right ventricle to pulmonary artery conduit.

